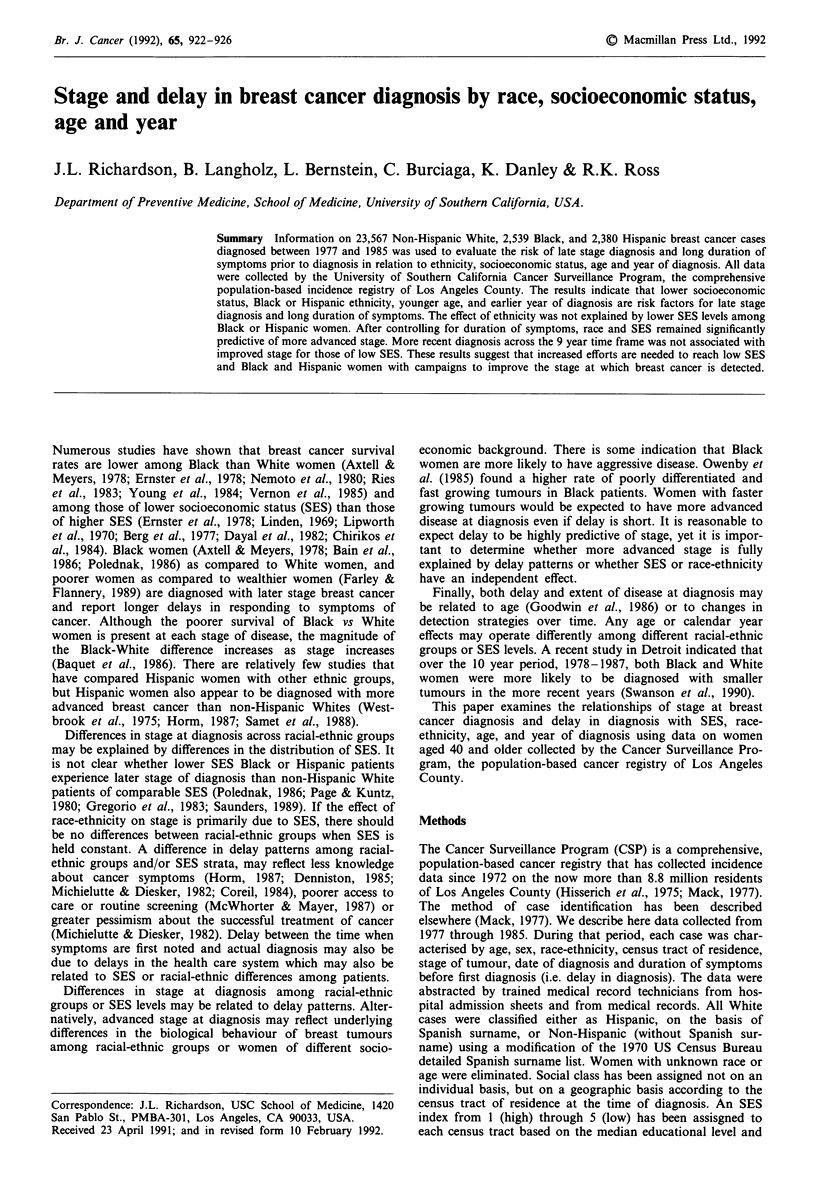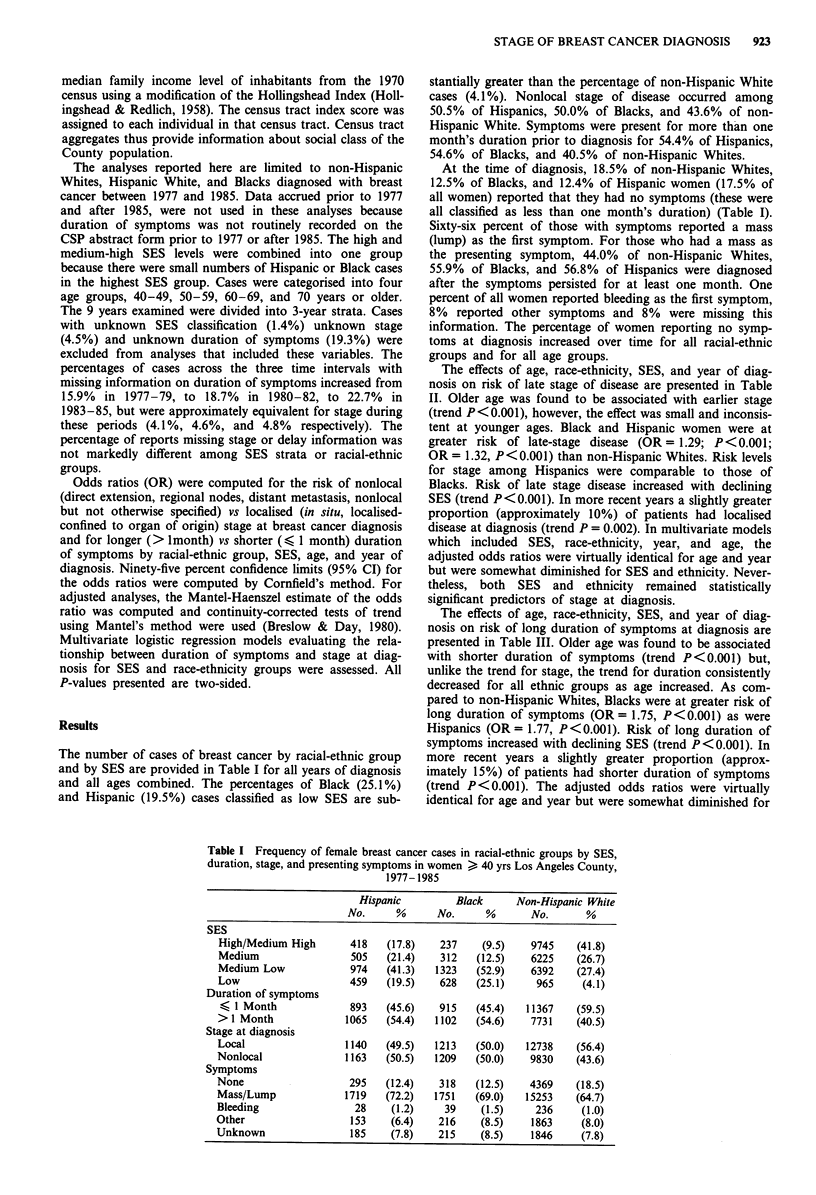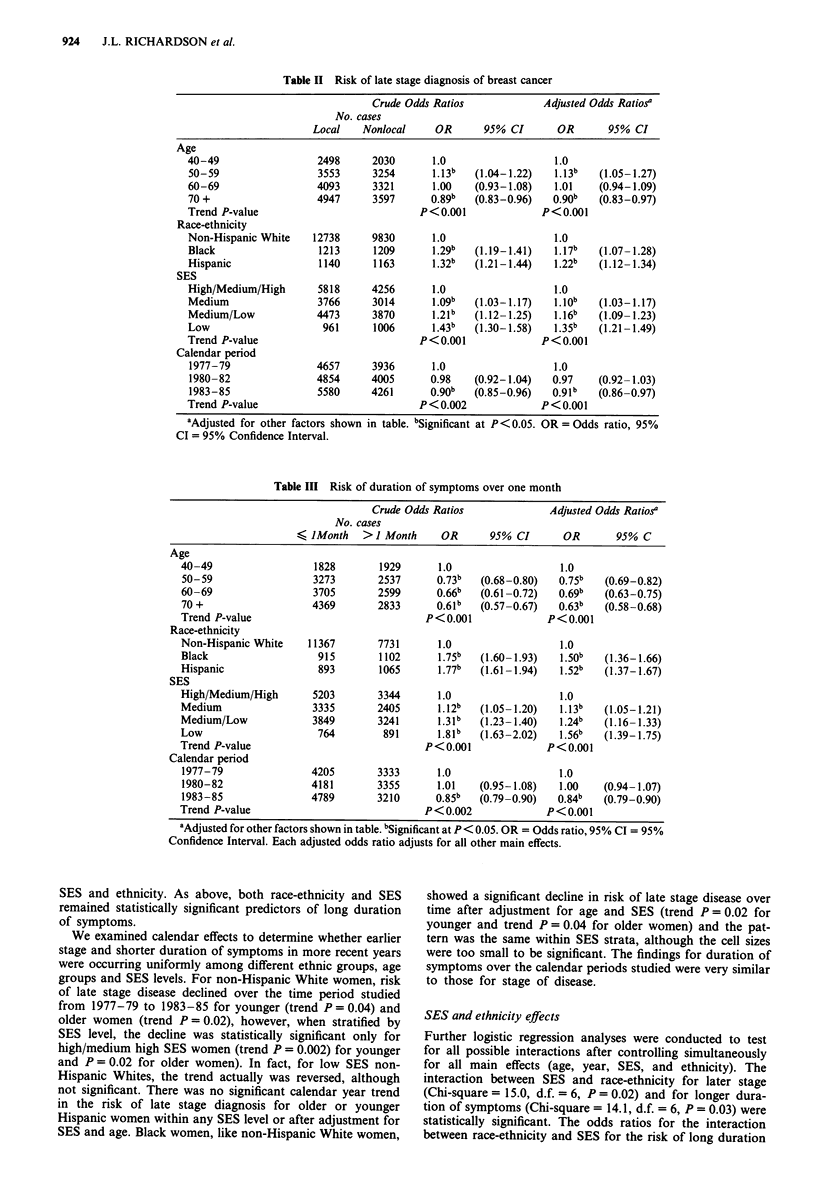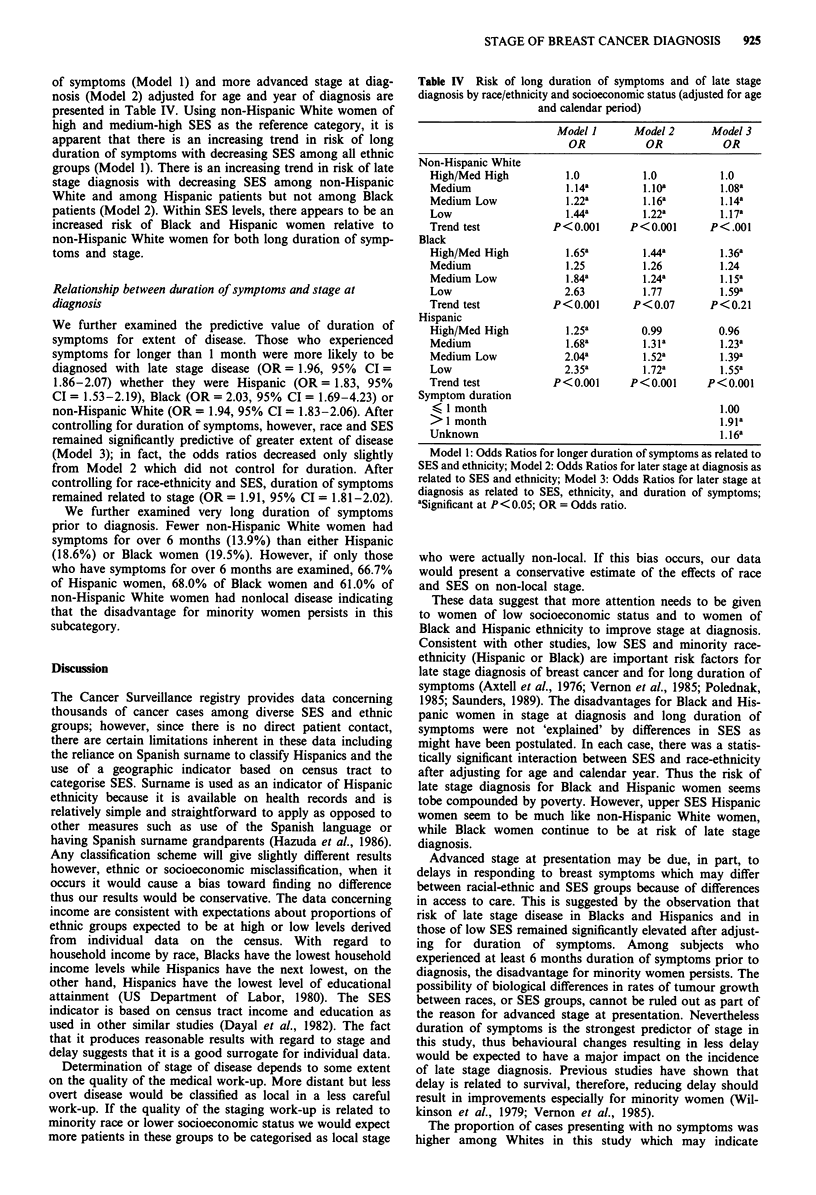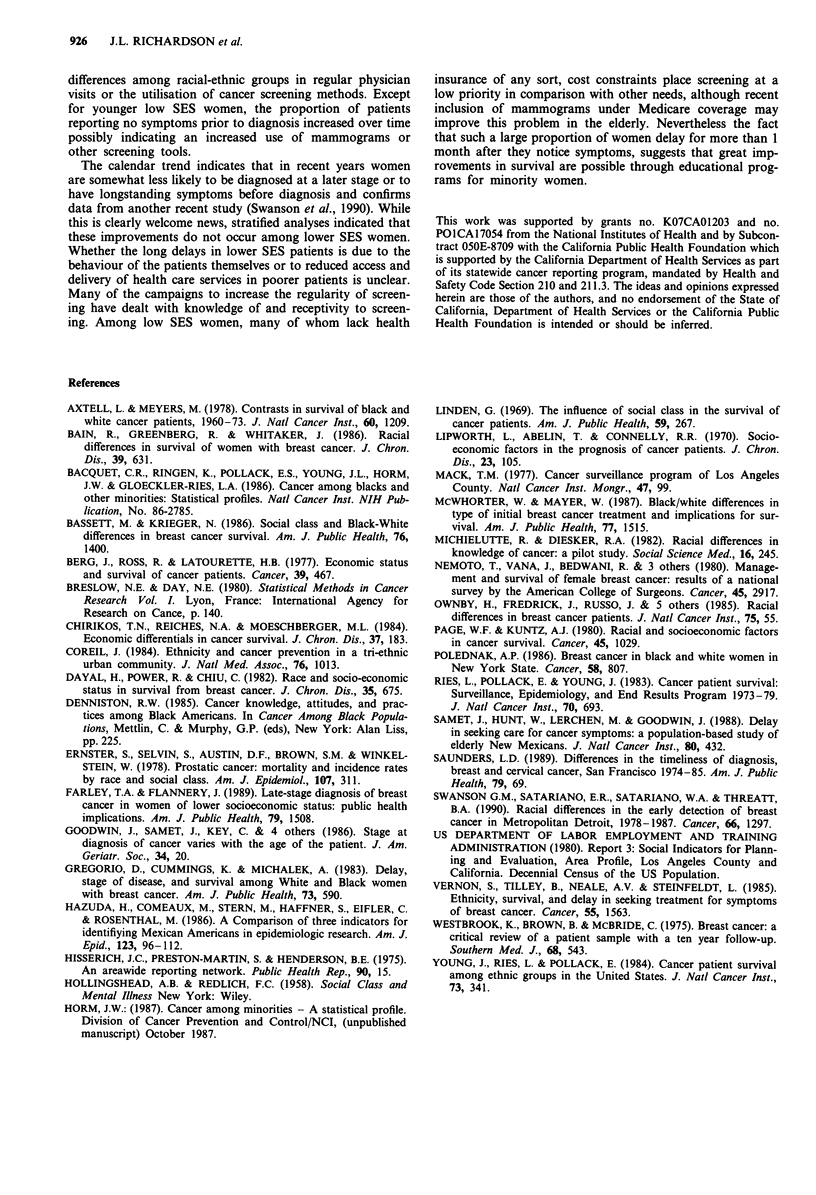# Stage and delay in breast cancer diagnosis by race, socioeconomic status, age and year.

**DOI:** 10.1038/bjc.1992.193

**Published:** 1992-06

**Authors:** J. L. Richardson, B. Langholz, L. Bernstein, C. Burciaga, K. Danley, R. K. Ross

**Affiliations:** Department of Preventive Medicine, School of Medicine, University of Southern California, Los Angeles 90033.

## Abstract

Information on 23,567 Non-Hispanic White, 2,539 Black, and 2,380 Hispanic breast cancer cases diagnosed between 1977 and 1985 was used to evaluate the risk of late stage diagnosis and long duration of symptoms prior to diagnosis in relation to ethnicity, socioeconomic status, age and year of diagnosis. All data were collected by the University of Southern California Cancer Surveillance Program, the comprehensive population-based incidence registry of Los Angeles County. The results indicate that lower socioeconomic status, Black or Hispanic ethnicity, younger age, and earlier year of diagnosis are risk factors for late stage diagnosis and long duration of symptoms. The effect of ethnicity was not explained by lower SES levels among Black or Hispanic women. After controlling for duration of symptoms, race and SES remained significantly predictive of more advanced stage. More recent diagnosis across the 9 year time frame was not associated with improved stage for those of low SES. These results suggest that increased efforts are needed to reach low SES and Black and Hispanic women with campaigns to improve the stage at which breast cancer is detected.


Br. J. Cancer (1992), 65, 922-926 © Macmillan Press Ltd., 1992

### Stage and delay in breast cancer diagnosis by race, socioeconomic status, age and year

J.L. Richardson, B. Langholz, L. Bernstein, C. Burciaga, K. Danley & R.K. Ross

*Department of Preventive Medicine, School of Medicine, University of Southern California, USA.*

**Summary** Information on 23,567 Non-Hispanic White, 2,539 Black, and 2,380 Hispanic breast cancer cases diagnosed between 1977 and 1985 was used to evaluate the risk of late stage diagnosis and long duration of symptoms prior to diagnosis in relation to ethnicity, socioeconomic status, age and year of diagnosis. All data were collected by the University of Southern California Cancer Surveillance Program, the comprehensive population-based incidence registry of Los Angeles County. The results indicate that lower socioeconomic status, Black or Hispanic ethnicity, younger age, and earlier year of diagnosis are risk factors for late stage diagnosis and long duration of symptoms. The effect of ethnicity was not explained by lower SES levels among Black or Hispanic women. After controlling for duration of symptoms, race and SES remained significantly predictive of more advanced stage. More recent diagnosis across the 9 year time frame was not associated with improved stage for those of low SES. These results suggest that increased efforts are needed to reach low SES and Black and Hispanic women with campaigns to improve the stage at which breast cancer is detected.

Numerous studies have shown that breast cancer survival rates are lower among Black than White women (Axtell & Meyers, 1978; Ernster *et al.*, 1978; Nemoto *et al.*, 1980; Ries *et al.*, 1983; Young *et al.*, 1984; Vernon *et al.*, 1985) and among those of lower socioeconomic status (SES) than those of higher SES (Ernster *et al.*, 1978; Linden, 1969; Lipworth *et al.*, 1970; Berg *et al.*, 1977; Dayal *et al.*, 1982; Chirikos *et al.*, 1984). Black women (Axtell & Meyers, 1978; Bain *et al.*, 1986; Polednak, 1986) as compared to White women, and poorer women as compared to wealthier women (Farley & Flannery, 1989) are diagnosed with later stage breast cancer and report longer delays in responding to symptoms of cancer. Although the poorer survival of Black *vs* White women is present at each stage of disease, the magnitude of the Black-White difference increases as stage increases (Baquet *et al.*, 1986). There are relatively few studies that have compared Hispanic women with other ethnic groups, but Hispanic women also appear to be diagnosed with more advanced breast cancer than non-Hispanic Whites (Westbrook *et al.*, 1975; Horm, 1987; Samet *et al.*, 1988).

Differences in stage at diagnosis across racial-ethnic groups may be explained by differences in the distribution of SES. It is not clear whether lower SES Black or Hispanic patients experience later stage of diagnosis than non-Hispanic White patients of comparable SES (Polednak, 1986; Page & Kuntz, 1980; Gregorio *et al.*, 1983; Saunders, 1989). If the effect of race-ethnicity on stage is primarily due to SES, there should be no differences between racial-ethnic groups when SES is held constant. A difference in delay patterns among racial-ethnic groups and/or SES strata, may reflect less knowledge about cancer symptoms (Horm, 1987; Denniston, 1985; Michielutte & Diesker, 1982; Coreil, 1984), poorer access to care or routine screening (McWhorter & Mayer, 1987) or greater pessimism about the successful treatment of cancer (Michielutte & Diesker, 1982). Delay between the time when symptoms are first noted and actual diagnosis may also be due to delays in the health care system which may also be related to SES or racial-ethnic differences among patients.

Differences in stage at diagnosis among racial-ethnic groups or SES levels may be related to delay patterns. Alternatively, advanced stage at diagnosis may reflect underlying differences in the biological behaviour of breast tumours among racial-ethnic groups or women of different socio-economic background. There is some indication that Black women are more likely to have aggressive disease. Owenby *et al.* (1985) found a higher rate of poorly differentiated and fast growing tumours in Black patients. Women with faster growing tumours would be expected to have more advanced disease at diagnosis even if delay is short. It is reasonable to expect delay to be highly predictive of stage, yet it is important to determine whether more advanced stage is fully explained by delay patterns or whether SES or race-ethnicity have an independent effect.

Finally, both delay and extent of disease at diagnosis may be related to age (Goodwin *et al.*, 1986) or to changes in detection strategies over time. Any age or calendar year effects may operate differently among different racial-ethnic groups or SES levels. A recent study in Detroit indicated that over the 10 year period, 1978-1987, both Black and White women were more likely to be diagnosed with smaller tumours in the more recent years (Swanson *et al.*, 1990).

This paper examines the relationships of stage at breast cancer diagnosis and delay in diagnosis with SES, race-ethnicity, age, and year of diagnosis using data on women aged 40 and older collected by the Cancer Surveillance Program, the population-based cancer registry of Los Angeles County.

### Methods

The Cancer Surveillance Program (CSP) is a comprehensive, population-based cancer registry that has collected incidence data since 1972 on the now more than 8.8 million residents of Los Angeles County (Hisserich *et al.*, 1975; Mack, 1977). The method of case identification has been described elsewhere (Mack, 1977). We describe here data collected from 1977 through 1985. During that period, each case was characterised by age, sex, race-ethnicity, census tract of residence, stage of tumour, date of diagnosis and duration of symptoms before first diagnosis (i.e. delay in diagnosis). The data were abstracted by trained medical record technicians from hospital admission sheets and from medical records. All White cases were classified either as Hispanic, on the basis of Spanish surname, or Non-Hispanic (without Spanish surname) using a modification of the 1970 US Census Bureau detailed Spanish surname list. Women with unknown race or age were eliminated. Social class has been assigned not on an individual basis, but on a geographic basis according to the census tract of residence at the time of diagnosis. An SES index from 1 (high) through 5 (low) has been assisgned to each census tract based on the median educational level and

Correspondence: J.L. Richardson, USC School of Medicine, 1420 San Pablo St., PMBA-301, Los Angeles, CA 90033, USA.
Received 23 April 1991; and in revised form 10 February 1992.

median family income level of inhabitants from the 1970 census using a modification of the Hollingshead Index (Hollingshead & Redlich, 1958). The census tract index score was assigned to each individual in that census tract. Census tract aggregates thus provide information about social class of the County population.

The analyses reported here are limited to non-Hispanic Whites, Hispanic White, and Blacks diagnosed with breast cancer between 1977 and 1985. Data accrued prior to 1977 and after 1985, were not used in these analyses because duration of symptoms was not routinely recorded on the CSP abstract form prior to 1977 or after 1985. The high and medium-high SES levels were combined into one group because there were small numbers of Hispanic or Black cases in the highest SES group. Cases were categorised into four age groups, 40–49, 50–59, 60–69, and 70 years or older. The 9 years examined were divided into 3-year strata. Cases with unknown SES classification (1.4%) unknown stage (4.5%) and unknown duration of symptoms (19.3%) were excluded from analyses that included these variables. The percentages of cases across the three time intervals with missing information on duration of symptoms increased from 15.9% in 1977–79, to 18.7% in 1980–82, to 22.7% in 1983–85, but were approximately equivalent for stage during these periods (4.1%, 4.6%, and 4.8% respectively). The percentage of reports missing stage or delay information was not markedly different among SES strata or racial-ethnic groups.

Odds ratios (OR) were computed for the risk of nonlocal (direct extension, regional nodes, distant metastasis, nonlocal but not otherwise specified) *vs* localised (*in situ*, localised-confined to organ of origin) stage at breast cancer diagnosis and for longer (> 1month) *vs* shorter (⩽ 1 month) duration of symptoms by racial-ethnic group, SES, age, and year of diagnosis. Ninety-five percent confidence limits (95% CI) for the odds ratios were computed by Cornfield's method. For adjusted analyses, the Mantel-Haenszel estimate of the odds ratio was computed and continuity-corrected tests of trend using Mantel's method were used (Breslow & Day, 1980). Multivariate logistic regression models evaluating the relationship between duration of symptoms and stage at diagnosis for SES and race-ethnicity groups were assessed. All *P*-values presented are two-sided.

### Results

The number of cases of breast cancer by racial-ethnic group and by SES are provided in Table I for all years of diagnosis and all ages combined. The percentages of Black (25.1%) and Hispanic (19.5%) cases classified as low SES are substantially greater than the percentage of non-Hispanic White cases (4.1%). Nonlocal stage of disease occurred among 50.5% of Hispanics, 50.0% of Blacks, and 43.6% of non-Hispanic White. Symptoms were present for more than one month's duration prior to diagnosis for 54.4% of Hispanics, 54.6% of Blacks, and 40.5% of non-Hispanic Whites.

At the time of diagnosis, 18.5% of non-Hispanic Whites, 12.5% of Blacks, and 12.4% of Hispanic women (17.5% of all women) reported that they had no symptoms (these were all classified as less than one month's duration) (Table I). Sixty-six percent of those with symptoms reported a mass (lump) as the first symptom. For those who had a mass as the presenting symptom, 44.0% of non-Hispanic Whites, 55.9% of Blacks, and 56.8% of Hispanics were diagnosed after the symptoms persisted for at least one month. One percent of all women reported bleeding as the first symptom, 8% reported other symptoms and 8% were missing this information. The percentage of women reporting no symptoms at diagnosis increased over time for all racial-ethnic groups and for all age groups.

The effects of age, race-ethnicity, SES, and year of diagnosis on risk of late stage of disease are presented in Table II. Older age was found to be associated with earlier stage (trend $P < 0.001$), however, the effect was small and inconsistent at younger ages. Black and Hispanic women were at greater risk of late-stage disease (OR = 1.29; $P < 0.001$; OR = 1.32, $P < 0.001$) than non-Hispanic Whites. Risk levels for stage among Hispanics were comparable to those of Blacks. Risk of late stage disease increased with declining SES (trend $P < 0.001$). In more recent years a slightly greater proportion (approximately 10%) of patients had localised disease at diagnosis (trend $P = 0.002$). In multivariate models which included SES, race-ethnicity, year, and age, the adjusted odds ratios were virtually identical for age and year but were somewhat diminished for SES and ethnicity. Nevertheless, both SES and ethnicity remained statistically significant predictors of stage at diagnosis.

The effects of age, race-ethnicity, SES, and year of diagnosis on risk of long duration of symptoms at diagnosis are presented in Table III. Older age was found to be associated with shorter duration of symptoms (trend $P < 0.001$) but, unlike the trend for stage, the trend for duration consistently decreased for all ethnic groups as age increased. As compared to non-Hispanic Whites, Blacks were at greater risk of long duration of symptoms (OR = 1.75, $P < 0.001$) as were Hispanics (OR = 1.77, $P < 0.001$). Risk of long duration of symptoms increased with declining SES (trend $P < 0.001$). In more recent years a slightly greater proportion (approximately 15%) of patients had shorter duration of symptoms (trend $P < 0.001$). The adjusted odds ratios were virtually identical for age and year but were somewhat diminished for

**Table I** Frequency of female breast cancer cases in racial-ethnic groups by SES, duration, stage, and presenting symptoms in women ⩾ 40 yrs Los Angeles County, 1977–1985

| | *Hispanic* | | *Black* | | *Non-Hispanic White* | |
|---|---|---|---|---|---|---|
| | *No.* | *%* | *No.* | *%* | *No.* | *%* |
| SES | | | | | | |
| High/Medium High | 418 | (17.8) | 237 | (9.5) | 9745 | (41.8) |
| Medium | 505 | (21.4) | 312 | (12.5) | 6225 | (26.7) |
| Medium Low | 974 | (41.3) | 1323 | (52.9) | 6392 | (27.4) |
| Low | 459 | (19.5) | 628 | (25.1) | 965 | (4.1) |
| Duration of symptoms | | | | | | |
| ⩽ 1 Month | 893 | (45.6) | 915 | (45.4) | 11367 | (59.5) |
| > 1 Month | 1065 | (54.4) | 1102 | (54.6) | 7731 | (40.5) |
| Stage at diagnosis | | | | | | |
| Local | 1140 | (49.5) | 1213 | (50.0) | 12738 | (56.4) |
| Nonlocal | 1163 | (50.5) | 1209 | (50.0) | 9830 | (43.6) |
| Symptoms | | | | | | |
| None | 295 | (12.4) | 318 | (12.5) | 4369 | (18.5) |
| Mass/Lump | 1719 | (72.2) | 1751 | (69.0) | 15253 | (64.7) |
| Bleeding | 28 | (1.2) | 39 | (1.5) | 236 | (1.0) |
| Other | 153 | (6.4) | 216 | (8.5) | 1863 | (8.0) |
| Unknown | 185 | (7.8) | 215 | (8.5) | 1846 | (7.8) |

**Table II** Risk of late stage diagnosis of breast cancer

| | No. cases | | Crude Odds Ratios | | Adjusted Odds Ratios[a] | |
|---|---|---|---|---|---|---|
| | *Local* | *Nonlocal* | *OR* | *95% CI* | *OR* | *95% CI* |
| Age | | | | | | |
| 40–49 | 2498 | 2030 | 1.0 | | 1.0 | |
| 50–59 | 3553 | 3254 | 1.13[b] | (1.04–1.22) | 1.13[b] | (1.05–1.27) |
| 60–69 | 4093 | 3321 | 1.00 | (0.93–1.08) | 1.01 | (0.94–1.09) |
| 70 + | 4947 | 3597 | 0.89[b] | (0.83–0.96) | 0.90[b] | (0.83–0.97) |
| Trend *P*-value | | | *P*<0.001 | | *P*<0.001 | |
| Race-ethnicity | | | | | | |
| Non-Hispanic White | 12738 | 9830 | 1.0 | | 1.0 | |
| Black | 1213 | 1209 | 1.29[b] | (1.19–1.41) | 1.17[b] | (1.07–1.28) |
| Hispanic | 1140 | 1163 | 1.32[b] | (1.21–1.44) | 1.22[b] | (1.12–1.34) |
| SES | | | | | | |
| High/Medium/High | 5818 | 4256 | 1.0 | | 1.0 | |
| Medium | 3766 | 3014 | 1.09[b] | (1.03–1.17) | 1.10[b] | (1.03–1.17) |
| Medium/Low | 4473 | 3870 | 1.21[b] | (1.12–1.25) | 1.16[b] | (1.09–1.23) |
| Low | 961 | 1006 | 1.43[b] | (1.30–1.58) | 1.35[b] | (1.21–1.49) |
| Trend *P*-value | | | *P*<0.001 | | *P*<0.001 | |
| Calendar period | | | | | | |
| 1977–79 | 4657 | 3936 | 1.0 | | 1.0 | |
| 1980–82 | 4854 | 4005 | 0.98 | (0.92–1.04) | 0.97 | (0.92–1.03) |
| 1983–85 | 5580 | 4261 | 0.90[b] | (0.85–0.96) | 0.91[b] | (0.86–0.97) |
| Trend *P*-value | | | *P*<0.002 | | *P*<0.001 | |

[a]Adjusted for other factors shown in table. [b]Significant at $P<0.05$. OR = Odds ratio, 95% CI = 95% Confidence Interval.

**Table III** Risk of duration of symptoms over one month

| | No. cases | | Crude Odds Ratios | | Adjusted Odds Ratios[a] | |
|---|---|---|---|---|---|---|
| | *≤ 1Month* | *> 1 Month* | *OR* | *95% CI* | *OR* | *95% C* |
| Age | | | | | | |
| 40–49 | 1828 | 1929 | 1.0 | | 1.0 | |
| 50–59 | 3273 | 2537 | 0.73[b] | (0.68–0.80) | 0.75[b] | (0.69–0.82) |
| 60–69 | 3705 | 2599 | 0.66[b] | (0.61–0.72) | 0.69[b] | (0.63–0.75) |
| 70 + | 4369 | 2833 | 0.61[b] | (0.57–0.67) | 0.63[b] | (0.58–0.68) |
| Trend *P*-value | | | *P*<0.001 | | *P*<0.001 | |
| Race-ethnicity | | | | | | |
| Non-Hispanic White | 11367 | 7731 | 1.0 | | 1.0 | |
| Black | 915 | 1102 | 1.75[b] | (1.60–1.93) | 1.50[b] | (1.36–1.66) |
| Hispanic | 893 | 1065 | 1.77[b] | (1.61–1.94) | 1.52[b] | (1.37–1.67) |
| SES | | | | | | |
| High/Medium/High | 5203 | 3344 | 1.0 | | 1.0 | |
| Medium | 3335 | 2405 | 1.12[b] | (1.05–1.20) | 1.13[b] | (1.05–1.21) |
| Medium/Low | 3849 | 3241 | 1.31[b] | (1.23–1.40) | 1.24[b] | (1.16–1.33) |
| Low | 764 | 891 | 1.81[b] | (1.63–2.02) | 1.56[b] | (1.39–1.75) |
| Trend *P*-value | | | *P*<0.001 | | *P*<0.001 | |
| Calendar period | | | | | | |
| 1977–79 | 4205 | 3333 | 1.0 | | 1.0 | |
| 1980–82 | 4181 | 3355 | 1.01 | (0.95–1.08) | 1.00 | (0.94–1.07) |
| 1983–85 | 4789 | 3210 | 0.85[b] | (0.79–0.90) | 0.84[b] | (0.79–0.90) |
| Trend *P*-value | | | *P*<0.002 | | *P*<0.001 | |

[a]Adjusted for other factors shown in table. [b]Significant at $P<0.05$. OR = Odds ratio, 95% CI = 95% Confidence Interval. Each adjusted odds ratio adjusts for all other main effects.

SES and ethnicity. As above, both race-ethnicity and SES remained statistically significant predictors of long duration of symptoms.

We examined calendar effects to determine whether earlier stage and shorter duration of symptoms in more recent years were occurring uniformly among different ethnic groups, age groups and SES levels. For non-Hispanic White women, risk of late stage disease declined over the time period studied from 1977–79 to 1983–85 for younger (trend $P = 0.04$) and older women (trend $P = 0.02$), however, when stratified by SES level, the decline was statistically significant only for high/medium high SES women (trend $P = 0.002$) for younger and $P = 0.02$ for older women). In fact, for low SES non-Hispanic Whites, the trend actually was reversed, although not significant. There was no significant calendar year trend in the risk of late stage diagnosis for older or younger Hispanic women within any SES level or after adjustment for SES and age. Black women, like non-Hispanic White women, showed a significant decline in risk of late stage disease over time after adjustment for age and SES (trend $P = 0.02$ for younger and trend $P = 0.04$ for older women) and the pattern was the same within SES strata, although the cell sizes were too small to be significant. The findings for duration of symptoms over the calendar periods studied were very similar to those for stage of disease.

### *SES and ethnicity effects*

Further logistic regression analyses were conducted to test for all possible interactions after controlling simultaneously for all main effects (age, year, SES, and ethnicity). The interaction between SES and race-ethnicity for later stage (Chi-square = 15.0, d.f. = 6, $P = 0.02$) and for longer duration of symptoms (Chi-square = 14.1, d.f. = 6, $P = 0.03$) were statistically significant. The odds ratios for the interaction between race-ethnicity and SES for the risk of long duration of symptoms (Model 1) and more advanced stage at diagnosis (Model 2) adjusted for age and year of diagnosis are presented in Table IV. Using non-Hispanic White women of high and medium-high SES as the reference category, it is apparent that there is an increasing trend in risk of long duration of symptoms with decreasing SES among all ethnic groups (Model 1). There is an increasing trend in risk of late stage diagnosis with decreasing SES among non-Hispanic White and among Hispanic patients but not among Black patients (Model 2). Within SES levels, there appears to be an increased risk of Black and Hispanic women relative to non-Hispanic White women for both long duration of symptoms and stage.

*Relationship between duration of symptoms and stage at diagnosis*

We further examined the predictive value of duration of symptoms for extent of disease. Those who experienced symptoms for longer than 1 month were more likely to be diagnosed with late stage disease (OR = 1.96, 95% CI = 1.86–2.07) whether they were Hispanic (OR = 1.83, 95% CI = 1.53–2.19), Black (OR = 2.03, 95% CI = 1.69–4.23) or non-Hispanic White (OR = 1.94, 95% CI = 1.83–2.06). After controlling for duration of symptoms, however, race and SES remained significantly predictive of greater extent of disease (Model 3); in fact, the odds ratios decreased only slightly from Model 2 which did not control for duration. After controlling for race-ethnicity and SES, duration of symptoms remained related to stage (OR = 1.91, 95% CI = 1.81–2.02).

We further examined very long duration of symptoms prior to diagnosis. Fewer non-Hispanic White women had symptoms for over 6 months (13.9%) than either Hispanic (18.6%) or Black women (19.5%). However, if only those who have symptoms for over 6 months are examined, 66.7% of Hispanic women, 68.0% of Black women and 61.0% of non-Hispanic White women had nonlocal disease indicating that the disadvantage for minority women persists in this subcategory.

**Table IV** Risk of long duration of symptoms and of late stage diagnosis by race/ethnicity and socioeconomic status (adjusted for age and calendar period)

| | *Model 1* *OR* | *Model 2* *OR* | *Model 3* *OR* |
|---|---|---|---|
| Non-Hispanic White | | | |
| High/Med High | 1.0 | 1.0 | 1.0 |
| Medium | 1.14[a] | 1.10[a] | 1.08[a] |
| Medium Low | 1.22[a] | 1.16[a] | 1.14[a] |
| Low | 1.44[a] | 1.22[a] | 1.17[a] |
| Trend test | $P<0.001$ | $P<0.001$ | $P<.001$ |
| Black | | | |
| High/Med High | 1.65[a] | 1.44[a] | 1.36[a] |
| Medium | 1.25 | 1.26 | 1.24 |
| Medium Low | 1.84[a] | 1.24[a] | 1.15[a] |
| Low | 2.63 | 1.77 | 1.59[a] |
| Trend test | $P<0.001$ | $P<0.07$ | $P<0.21$ |
| Hispanic | | | |
| High/Med High | 1.25[a] | 0.99 | 0.96 |
| Medium | 1.68[a] | 1.31[a] | 1.23[a] |
| Medium Low | 2.04[a] | 1.52[a] | 1.39[a] |
| Low | 2.35[a] | 1.72[a] | 1.55[a] |
| Trend test | $P<0.001$ | $P<0.001$ | $P<0.001$ |
| Symptom duration | | | |
| $\leqslant$ 1 month | | | 1.00 |
| $>$ 1 month | | | 1.91[a] |
| Unknown | | | 1.16[a] |

Model 1: Odds Ratios for longer duration of symptoms as related to SES and ethnicity; Model 2: Odds Ratios for later stage at diagnosis as related to SES and ethnicity; Model 3: Odds Ratios for later stage at diagnosis as related to SES, ethnicity, and duration of symptoms; [a]Significant at $P<0.05$; OR = Odds ratio.

### Discussion

The Cancer Surveillance registry provides data concerning thousands of cancer cases among diverse SES and ethnic groups; however, since there is no direct patient contact, there are certain limitations inherent in these data including the reliance on Spanish surname to classify Hispanics and the use of a geographic indicator based on census tract to categorise SES. Surname is used as an indicator of Hispanic ethnicity because it is available on health records and is relatively simple and straightforward to apply as opposed to other measures such as use of the Spanish language or having Spanish surname grandparents (Hazuda *et al.*, 1986). Any classification scheme will give slightly different results however, ethnic or socioeconomic misclassification, when it occurs it would cause a bias toward finding no difference thus our results would be conservative. The data concerning income are consistent with expectations about proportions of ethnic groups expected to be at high or low levels derived from individual data on the census. With regard to household income by race, Blacks have the lowest household income levels while Hispanics have the next lowest, on the other hand, Hispanics have the lowest level of educational attainment (US Department of Labor, 1980). The SES indicator is based on census tract income and education as used in other similar studies (Dayal *et al.*, 1982). The fact that it produces reasonable results with regard to stage and delay suggests that it is a good surrogate for individual data.

Determination of stage of disease depends to some extent on the quality of the medical work-up. More distant but less overt disease would be classified as local in a less careful work-up. If the quality of the staging work-up is related to minority race or lower socioeconomic status we would expect more patients in these groups to be categorised as local stage who were actually non-local. If this bias occurs, our data would present a conservative estimate of the effects of race and SES on non-local stage.

These data suggest that more attention needs to be given to women of low socioeconomic status and to women of Black and Hispanic ethnicity to improve stage at diagnosis. Consistent with other studies, low SES and minority race-ethnicity (Hispanic or Black) are important risk factors for late stage diagnosis of breast cancer and for long duration of symptoms (Axtell *et al.*, 1976; Vernon *et al.*, 1985; Polednak, 1985; Saunders, 1989). The disadvantages for Black and Hispanic women in stage at diagnosis and long duration of symptoms were not ‘explained’ by differences in SES as might have been postulated. In each case, there was a statistically significant interaction between SES and race-ethnicity after adjusting for age and calendar year. Thus the risk of late stage diagnosis for Black and Hispanic women seems tobe compounded by poverty. However, upper SES Hispanic women seem to be much like non-Hispanic White women, while Black women continue to be at risk of late stage diagnosis.

Advanced stage at presentation may be due, in part, to delays in responding to breast symptoms which may differ between racial-ethnic and SES groups because of differences in access to care. This is suggested by the observation that risk of late stage disease in Blacks and Hispanics and in those of low SES remained significantly elevated after adjusting for duration of symptoms. Among subjects who experienced at least 6 months duration of symptoms prior to diagnosis, the disadvantage for minority women persists. The possibility of biological differences in rates of tumour growth between races, or SES groups, cannot be ruled out as part of the reason for advanced stage at presentation. Nevertheless duration of symptoms is the strongest predictor of stage in this study, thus behavioural changes resulting in less delay would be expected to have a major impact on the incidence of late stage diagnosis. Previous studies have shown that delay is related to survival, therefore, reducing delay should result in improvements especially for minority women (Wilkinson *et al.*, 1979; Vernon *et al.*, 1985).

The proportion of cases presenting with no symptoms was higher among Whites in this study which may indicate differences among racial-ethnic groups in regular physician visits or the utilisation of cancer screening methods. Except for younger low SES women, the proportion of patients reporting no symptoms prior to diagnosis increased over time possibly indicating an increased use of mammograms or other screening tools.

The calendar trend indicates that in recent years women are somewhat less likely to be diagnosed at a later stage or to have longstanding symptoms before diagnosis and confirms data from another recent study (Swanson *et al.*, 1990). While this is clearly welcome news, stratified analyses indicated that these improvements do not occur among lower SES women. Whether the long delays in lower SES patients is due to the behaviour of the patients themselves or to reduced access and delivery of health care services in poorer patients is unclear. Many of the campaigns to increase the regularity of screening have dealt with knowledge of and receptivity to screening. Among low SES women, many of whom lack health insurance of any sort, cost constraints place screening at a low priority in comparison with other needs, although recent inclusion of mammograms under Medicare coverage may improve this problem in the elderly. Nevertheless the fact that such a large proportion of women delay for more than 1 month after they notice symptoms, suggests that great improvements in survival are possible through educational programs for minority women.

This work was supported by grants no. K07CA01203 and no. PO1CA17054 from the National Institutes of Health and by Subcontract 050E-8709 with the California Public Health Foundation which is supported by the California Department of Health Services as part of its statewide cancer reporting program, mandated by Health and Safety Code Section 210 and 211.3. The ideas and opinions expressed herein are those of the authors, and no endorsement of the State of California, Department of Health Services or the California Public Health Foundation is intended or should be inferred.

### References

AXTELL, L. & MEYERS, M. (1978). Contrasts in survival of black and white cancer patients, 1960–73. *J. Natl Cancer Inst.*, **60**, 1209.

BAIN, R., GREENBERG, R. & WHITAKER, J. (1986). Racial differences in survival of women with breast cancer. *J. Chron. Dis.*, **39**, 631.

BACQUET, C.R., RINGEN, K., POLLACK, E.S., YOUNG, J.L., HORM, J.W. & GLOECKLER-RIES, L.A. (1986). Cancer among blacks and other minorities: Statistical profiles. *Natl Cancer Inst. NIH Publication*, No. 86-2785.

BASSETT, M. & KRIEGER, N. (1986). Social class and Black-White differences in breast cancer survival. *Am. J. Public Health*, **76**, 1400.

BERG, J., ROSS, R. & LATOURETTE, H.B. (1977). Economic status and survival of cancer patients. *Cancer*, **39**, 467.

BRESLOW, N.E. & DAY, N.E. (1980). *Statistical Methods in Cancer Research Vol. I*. Lyon, France: International Agency for Research on Cance, p. 140.

CHIRIKOS, T.N., REICHES, N.A. & MOESCHBERGER, M.L. (1984). Economic differentials in cancer survival. *J. Chron. Dis.*, **37**, 183.

COREIL, J. (1984). Ethnicity and cancer prevention in a tri-ethnic urban community. *J. Natl Med. Assoc.*, **76**, 1013.

DAYAL, H., POWER, R. & CHIU, C. (1982). Race and socio-economic status in survival from breast cancer. *J. Chron. Dis.*, **35**, 675.

DENNISTON, R.W. (1985). Cancer knowledge, attitudes, and practices among Black Americans. In *Cancer Among Black Populations*, Mettlin, C. & Murphy, G.P. (eds), New York: Alan Liss, pp. 225.

ERNSTER, S., SELVIN, S., AUSTIN, D.F., BROWN, S.M. & WINKELSTEIN, W. (1978). Prostatic cancer: mortality and incidence rates by race and social class. *Am. J. Epidemiol.*, **107**, 311.

FARLEY, T.A. & FLANNERY, J. (1989). Late-stage diagnosis of breast cancer in women of lower socioeconomic status: public health implications. *Am. J. Public Health*, **79**, 1508.

GOODWIN, J., SAMET, J., KEY, C. & 4 others (1986). Stage at diagnosis of cancer varies with the age of the patient. *J. Am. Geriatr. Soc.*, **34**, 20.

GREGORIO, D., CUMMINGS, K. & MICHALEK, A. (1983). Delay, stage of disease, and survival among White and Black women with breast cancer. *Am. J. Public Health*, **73**, 590.

HAZUDA, H., COMEAUX, M., STERN, M., HAFFNER, S., EIFLER, C. & ROSENTHAL, M. (1986). A Comparison of three indicators for identifiying Mexican Americans in epidemiologic research. *Am. J. Epid.*, **123**, 96–112.

HISSERICH, J.C., PRESTON-MARTIN, S. & HENDERSON, B.E. (1975). An areawide reporting network. *Public Health Rep.*, **90**, 15.

HOLLINGSHEAD, A.B. & REDLICH, F.C. (1958). *Social Class and Mental Illness* New York: Wiley.

HORM, J.W.: (1987). Cancer among minorities – A statistical profile. Division of Cancer Prevention and Control/NCI, (unpublished manuscript) October 1987.

LINDEN, G. (1969). The influence of social class in the survival of cancer patients. *Am. J. Public Health*, **59**, 267.

LIPWORTH, L., ABELIN, T. & CONNELLY, R.R. (1970). Socioeconomic factors in the prognosis of cancer patients. *J. Chron. Dis.*, **23**, 105.

MACK, T.M. (1977). Cancer surveillance program of Los Angeles County. *Natl Cancer Inst. Mongr.*, **47**, 99.

McWHORTER, W. & MAYER, W. (1987). Black/white differences in type of initial breast cancer treatment and implications for survival. *Am. J. Public Health*, **77**, 1515.

MICHIELUTTE, R. & DIESKER, R.A. (1982). Racial differences in knowledge of cancer: a pilot study. *Social Science Med.*, **16**, 245.

NEMOTO, T., VANA, J., BEDWANI, R. & 3 others (1980). Management and survival of female breast cancer: results of a national survey by the American College of Surgeons. *Cancer*, **45**, 2917.

OWNBY, H., FREDRICK, J., RUSSO, J. & 5 others (1985). Racial differences in breast cancer patients. *J. Natl Cancer Inst.*, **75**, 55.

PAGE, W.F. & KUNTZ, A.J. (1980). Racial and socioeconomic factors in cancer survival. *Cancer*, **45**, 1029.

POLEDNAK, A.P. (1986). Breast cancer in black and white women in New York State. *Cancer*, **58**, 807.

RIES, L., POLLACK, E. & YOUNG, J. (1983). Cancer patient survival: Surveillance, Epidemiology, and End Results Program 1973–79. *J. Natl Cancer Inst.*, **70**, 693.

SAMET, J., HUNT, W., LERCHEN, M. & GOODWIN, J. (1988). Delay in seeking care for cancer symptoms: a population-based study of elderly New Mexicans. *J. Natl Cancer Inst.*, **80**, 432.

SAUNDERS, L.D. (1989). Differences in the timeliness of diagnosis, breast and cervical cancer, San Francisco 1974–85. *Am. J. Public Health*, **79**, 69.

SWANSON G.M., SATARIANO, E.R., SATARIANO, W.A. & THREATT, B.A. (1990). Racial differences in the early detection of breast cancer in Metropolitan Detroit, 1978–1987. *Cancer*, **66**, 1297.

US DEPARTMENT OF LABOR EMPLOYMENT AND TRAINING ADMINISTRATION (1980). Report 3: Social Indicators for Planning and Evaluation, Area Profile, Los Angeles County and California. Decennial Census of the US Population.

VERNON, S., TILLEY, B., NEALE, A.V. & STEINFELDT, L. (1985). Ethnicity, survival, and delay in seeking treatment for symptoms of breast cancer. *Cancer*, **55**, 1563.

WESTBROOK, K., BROWN, B. & McBRIDE, C. (1975). Breast cancer: a critical review of a patient sample with a ten year follow-up. *Southern Med. J.*, **68**, 543.

YOUNG, J., RIES, L. & POLLACK, E. (1984). Cancer patient survival among ethnic groups in the United States. *J. Natl Cancer Inst.*, **73**, 341.